# Oxidative Stress and Hemoglobin Level of Complicated and Uncomplicated Malaria Cases among Children: A Cross-Sectional Study in Kumasi Metropolis, Ghana

**DOI:** 10.1155/2019/8479076

**Published:** 2019-07-07

**Authors:** Kwabena Nsiah, Bernard Bahaah, Bright Oppong Afranie, Simon Koffie, Emmanuel Akowuah, Sampson Donkor

**Affiliations:** ^1^Department of Biochemistry and Biotechnology, Faculty of Biosciences, Kwame Nkrumah University of Science and Technology, Kumasi, Ghana; ^2^Department of Molecular Medicine, School of Medicine and Dentistry, Kwame Nkrumah University of Science and Technology, Kumasi, Ghana; ^3^Kumasi Centre for Collaborative Research in Tropical Medicine, Kwame Nkrumah University of Science and Technology, Kumasi, Ghana

## Abstract

**Introduction:**

Malaria is a leading cause of mortality among children below 5 years in Ghana. Its parasites are known to cause the degradation of hemoglobin, resulting in the production of reactive oxygen species and hence oxidant stress. Therefore, this study was carried out to compare the levels of oxidative stress between children with complicated and uncomplicated malaria infection in Kumasi, Ghana.

**Method:**

Subjects were recruited from hospitals in the Kumasi Metropolis. This was a cross-sectional study, involving 17 complicated malaria subjects, 51 uncomplicated malaria subjects, and 15 nonparasitemic subjects. The rapid diagnostic test (RDT) was used to determine presence or absence of* falciparum* malaria among the study participants. Blood samples from subjects were used to determine hemoglobin, malondialdehyde (MDA), and vitamin C levels.

**Results:**

Majority of the subjects (67.5%) were within the age of 0-5 years. The mean age (±SD) of uncomplicated malaria subjects was 4.32 (±2.81) years, while that of complicated malaria was 4.27 (±2.96). Mean levels of HB decreased significantly in the following order: control subjects > uncomplicated malaria subjects > complicated malaria subjects (p<0.0001). Mean levels of MDA were significantly lower in control subjects compared to complicated malaria subjects (4.62±1.85 versus 6.68±0.70, p=0.0008) and also lowered in uncomplicated malaria subjects compared to complicated malaria (4.50±1.58 versus 6.68±0.70, p<0.0001). There was a statistically significant reduced mean level of vitamin C (p=0.036) in the following order: control subjects > uncomplicated malaria > complicated malaria subjects. However, for the complicated malaria cases, there were significantly higher mean vitamin C levels in females than in males (p<0.001).

**Conclusion:**

Malaria progression increases MDA levels and decreases the ascorbate (vitamin C) and hemoglobin levels. It is recommended that future studies should investigate changes in other antioxidant vitamins, like vitamins A and E.

## 1. Introduction

A report from World Health Organization (WHO) states that malaria is a significant global health problem in more than 100 countries, especially those in the tropics, and causes more than 500,000 deaths annually. Over 90% of these deaths occur in sub-Saharan Africa, where the disease is estimated to kill one child every 30 seconds [1]. In the first quarter of 2015, Ghana recorded about 2.2 million suspected cases of malaria, which represent a 3.50% increase over 2014 [2]. Basically malaria symptoms can be classified into two categories, uncomplicated and severe (complicated) cases [3], and are attributed to the infection caused by malaria parasites in the host.

The natural host defence mechanism is activated with involvement of phagocytes (macrophages and neutrophils) in response to infection caused by* Plasmodium* parasites [4]. The parasites that invade the red cells also cause the breakdown of hemoglobin. This results in the generation of large amounts of reactive oxygen species (ROS) and reactive nitrogen species (RNS), causing a disparity between the activity of antioxidants and formation of oxidizing species. This disparity triggers oxidative stress, which is an important mechanism of human hosts, in response to infections [5]. The role of oxidative stress during malaria infection is still unclear. Some authors suggest a protective role, whereas others ascribe a detrimental role, driving the pathophysiology of the disease [6]. A study by Guha and colleagues (2006) suggested that the generation of reactive oxygen and nitrogen species plays a crucial role in the development of systemic complications caused by malaria [7].

Oxidative stress markers such as malondialdehyde levels in persons with malaria are higher, compared to the uninfected ones, while catalase activities are lower in those with malaria than those without the disease [8]. Oxidative stress results from increased production of free radicals, causing increased formation of malondialdehyde (MDA), an important lipid peroxidation marker [9]. However, ascorbic acid (vitamin C), which has antioxidant property, is reported to mop up free radicals. Since malaria infection imposes tremendous oxidative stress on the host, there is a need to investigate the gap between complicated and uncomplicated malaria, especially among children [10].

Information on the level of oxidative stress during* Plasmodium falciparum* infection among Ghanaian children is sparse and, against this background, this study sought to determine the oxidative stress level of complicated and uncomplicated malaria cases among some children in Kumasi, Ghana. This will help in finding ways to counteract the oxidative stress, which accompanies the progression of uncomplicated malaria to complicated malaria.

## 2. Method

### 2.1. Study Design/Study Site

The study was a cross-sectional one, carried out from February 2017 to June 2017 in four selected hospitals in Kumasi, Ashanti Region, Ghana. These were Maternal and Child Health Hospital, Manhyia District Hospital, Suntreso Government Hospital, and KNUST Hospital. Kumasi is a malaria-endemic zone (https://www.againstmalaria.com/Distribution.aspx?proposalID=42. 26th June, 2017) and has a total population of 2,069,350.

### 2.2. Study Population

All children below twelve years who reported to these facilities with symptoms of malaria were screened for this study. Children who visited the hospitals and tested negative to malaria parasites were recruited into this study as controls. Participants positive to malaria parasites were classified as cases and, based on their hemoglobin and hematocrit levels, the cases were categorized into complicated and uncomplicated malaria patients. Children who reported to these hospitals with conditions like human immunodeficiency virus (HIV), enteric fever, sickle cell disease, hepatitis B and C infections, and pyrexia of unknown origin were not included in the study. The uncomplicated malaria patients were those who reported with the usual symptoms of malaria but were not admitted in the wards of these hospitals and did not have any malaria complication. The complicated patients or complicated malaria group were those who were admitted in the hospital wards and, in addition, had one or more of the complications specified by WHO (2014) to be present in these patients. Study participants with hemoglobin level below 11 g/dl were classified as anemic [11].

### 2.3. Definition of Complicated Malaria

World Health Organization (2014) defines complicated malaria in children as the presence of* P. falciparum* but no other confirmed cause for signs and symptoms and vital organ dysfunction with clinical features such as impaired consciousness, prostration, multiple convulsions, acidotic breathing, acute pulmonary edema and acute respiratory distress, shock, acute kidney injury, and/or clinical jaundice plus one or more of laboratory findings such as hypoglycemia (less than 2.2 mmol/L), metabolic acidosis (plasma bicarbonate less than 5 g/dl), severe normocytic anemia (less than 5 g/dl and packed cell volume less than 15%), hemoglobinuria, hyperlactatemia (greater than 5 mmol/L), pulmonary edema (radiological), and renal impairment (serum creatinine greater than 265 *μ*mol/L). Modifications were made where necessary to suit this study. Complicated malaria was defined in this study as the presence of* P. falciparum* asexual parasitemia and the absence of an identified cause: hemoglobin concentration below 5 g/dl or a hematocrit of less than 15% in children below 12 years of age [12].

### 2.4. Ethical Considerations

Ethical clearance for the study was obtained from the Committee on Human Research, Publication and Ethics (CHRPE) at the KNUST School of Medical Sciences/Komfo Anokye Teaching Hospital in Kumasi, Ghana (reference number: CHRPE/AP/078/17). Permission was also given by the various Medical Directors/Superintendents or Heads of Departments of the study facilities. Consent was also obtained from guardians or parents whose wards were recruited into this study.

### 2.5. Data Collection

A questionnaire was used to obtain information on demographic data of the subjects from their guardians. The rapid diagnostic test (RDT) was used to determine presence or absence of* P. falciparum* malaria. Of the 5 ml of venous blood collected, 3 ml was kept in a vacutainer plain tube and 2 ml in an EDTA tube. The clotted samples in the vacutainer tubes were centrifuged at 1500 g for 5-10 min and the serum was stored at −20°C until being assayed. The sample in the EDTA tube was used to determine the level of hemoglobin in the patients.

### 2.6. Measurement of Hemoglobin Levels

Hemoglobin level of each blood sample in EDTA tube was determined using an automated hematological analyzer (Sysmex Automated Hematology Analyzer, Kobe, Japan, XP-300, model no.: AC580857).

### 2.7. Vitamin C and MDA Determination Using ELISA

The malondialdehyde concentration (MDA), as well as vitamin C level, was measured, using the serum sample. ELISA kits obtained from Biobase Biotech, Shanghai, China, were used. The protocols for determining vitamin C and MDA were followed. The concentrations of MDA and vitamin C were finally measured spectrophotometrically, using an ELISA microplate analyzer (inqaba biotec, UK, model no.: RT0400814GDM).

### 2.8. Data Analysis

Data were entered into Microsoft Excel 2013 and cleaned before being subjected to analysis. About 8 of the study participants were having missing data and so were excluded from the study. Statistical analyses were performed using GraphPad Prism 6 and SPSS version 20.0. Means were calculated for continuous variables, while percentages were calculated for categorical variables. Data presentation was done by using tables and bar graphs.* t*-test was used when comparing a pair of distributions and ANOVA/Tukey test was used when comparing more than two distributions. A p value < 0.05 was considered statistically significant.

## 3. Results

### 3.1. Ages and Gender of Study Participants

There were 17 subjects with severe or complicated malaria, 15 control subjects, and 51 subjects with uncomplicated malaria recruited into the study. The mean age (±SD) of uncomplicated malaria subjects was 4.32 (±2.81), while that of complicated malaria stood at 4.27 (±2.96).

### 3.2. Hemoglobin, Serum MDA, and Vitamin C Levels of Study Participants according to Gender

Table 2 shows that there was no significant difference between males and females in uncomplicated malaria for mean levels of HB (p=0.391), vitamin C (p=0.310), and MDA (p=0.528). However, for the complicated malaria cases, there was significant difference as mean value of serum vitamin C levels was lower in males than in females (p<0.001).

### 3.3. Correlation Analysis of MDA and Vitamin C against Hemoglobin Level among Participants

Correlation analysis of the biomarkers of oxidative stress was done against hemoglobin levels. MDA levels were negatively correlated with hemoglobin levels, while vitamin C levels were positively correlated with hemoglobin levels, even though they were not statistically significant. (Table 3).

**Figure 1:**
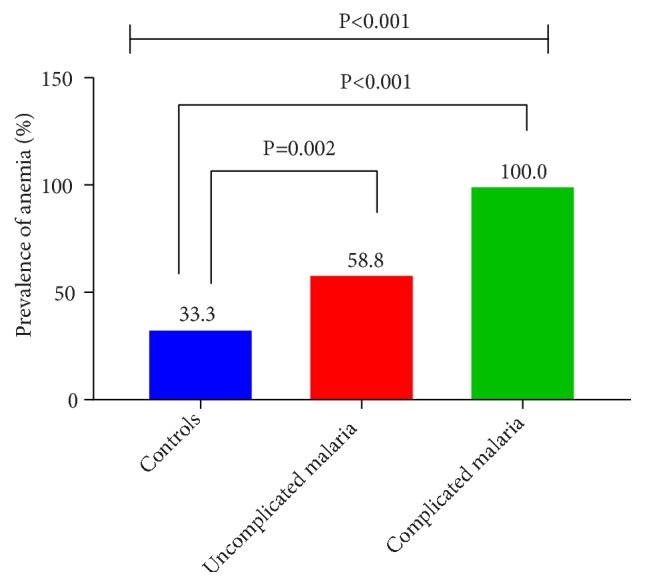
Prevalence of anemia among control, uncomplicated, and complicated malaria cases.

For the patients with uncomplicated malaria, 58.8% were anemic; all the patients with complicated malaria were anemic, while 33.3% of the control were anemic and there were significant differences in the proportions in various groups as shown in Figure 1.

Table 4 shows the comparison of hemoglobin, serum MDA, and vitamin C levels among control, uncomplicated, and complicated malaria subjects. Mean levels of HB decreased significantly in the following order: control subjects > uncomplicated malaria subjects > complicated malaria subjects (p<0.0001). Mean levels of MDA were significantly lower in control subjects compared to complicated malaria subjects (4.62±1.85 versus 6.68±0.70, p=0.0008) and also lowered in uncomplicated malaria compared to complicated malaria (4.50±1.58 versus 6.68±0.70, p<0.0001). There were statistically significantly reduced mean levels of vitamin C (p=0.036) in the following order: control subjects > uncomplicated malaria subjects > complicated malaria subjects.

**Figure 2:**
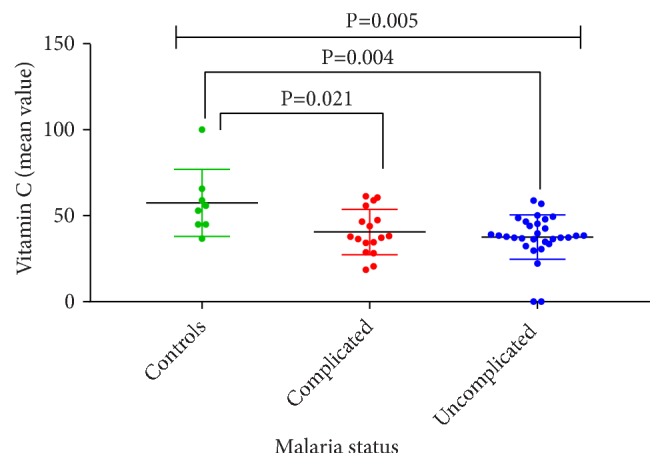
Comparison of vitamin C of the anemic control subjects and anemic uncomplicated and complicated malaria subjects.

From Figure 2, mean level of vitamin C was higher among the anemic control, anemic uncomplicated malaria, and anemic complicated malaria subjects. The mean levels of vitamin C were higher in anemic control subjects compared to anemic uncomplicated ones (54.0±19.0 versus 38.2±13.7, p=0.004) and anemic complicated malaria subjects (39.5±9.9, p=0.021). However, no significant difference was observed between anemic uncomplicated and complicated malaria subjects (38.2±13.7 versus 39.5±9.9, p=0.942).

**Figure 3:**
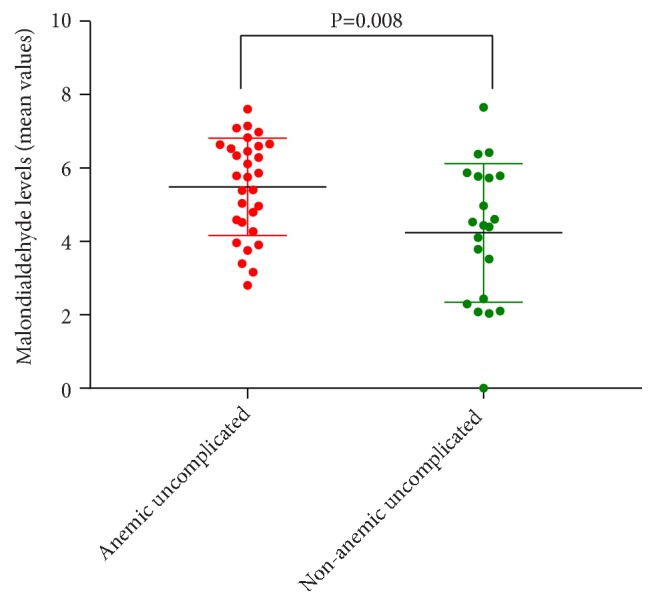
Comparison of malondialdehyde levels between anemic and nonanemic subjects with uncomplicated malaria.

Higher mean level of malondialdehyde was observed in the anemic subjects, compared to the nonanemic subjects with uncomplicated malaria, but this was statistically significant (5.17±1.69 versus 4.34±1.70, p=0.008) as shown in Figure 3.

## 4. Discussion

Malaria activates the immune system of the body, thereby causing release of reactive oxygen species (ROS) and reactive nitrogen species (RNS), which leads to degradation of hemoglobin (Kulkarni et al., 2003). When malaria is not treated early, it may progress to the severe form, which is highly lethal. Severe malaria is a major cause of morbidity and mortality in children in developing countries, including Ghana [8].

In Ghana, malaria is estimated to cause 8% of all certified deaths and ranks as the commonest cause of death in children below 5 years of age [2]. The study revealed that children mostly affected by malaria are those below five years (Table 1). The mean ages for both uncomplicated malaria subjects (4.32±2.81) and complicated malaria subjects (4.27±2.96) were very close. This was not surprising as uncomplicated malaria when not treated early advances to the severe form in a matter of months. It has been reported elsewhere that most malaria infections and deaths occur within this age group [13, 14]. The main reason for this is their immature or weak immune system [12]. As has been indicated earlier, malaria can easily progress to the severe form when treatment is delayed. As the disease severity progresses, the level of stress within the patients also increases. One simple way to monitor malaria disease severity is to monitor the concentration of malondialdehyde (MDA) [15].

The study by Narsaria* et al*. (2011) [15] revealed significant increase in MDA but decrease in ascorbic acid and other hematological and biochemical parameters during complicated malaria infection. In the current study, there was no significant difference between males and females with uncomplicated malaria, in relation to hemoglobin, MDA, and vitamin C. This implies that the levels of disease severity were similar in both sexes. However, with disease progression, depending on an individual patient and the patient's circumstances, there could be varied alterations occurring in some of these parameters [15]. This is exemplified by the female subjects with complicated malaria having significantly (p<0.001) higher levels of vitamin C, compared to the male subjects.

It is known that a strong antioxidant system will prevent excessive formation of MDA. Therefore, it is not surprising that patients with higher vitamin C (antioxidant vitamin) had lower MDA values. Mean level of MDA was significantly lower in control subjects, compared to complicated malaria subjects (p<0.001), which agrees with a study by Sakyi and colleagues (2012). The increased plasma MDA level shows lipid peroxidation due to the release of free radicals by the action of the malaria parasites [16]. There was a statistically significantly reduced mean level of vitamin C (p=0.036) in the following order: control > uncomplicated malaria > complicated malaria subjects. This observation agrees with other findings [15], implying that the more severe the disease gets, the lesser the concentration of ascorbate in the serum of the patient becomes. The prevalence of anemia among uncomplicated malaria patients stood at 58.8%, whereas that of the complicated or severe malaria cases was 100%. During the erythrocytic stage of the malaria parasite, important amino acids from hemoglobin are needed for the development and continuation of the life cycle of the parasite [17]. Thus, the presence of the parasites tends to facilitate the breakdown of the host's hemoglobin, through which the parasites derive their source of sustenance and growth, reflected in their ability to multiply. The extent of degradation of hemoglobin will be dependent on the severity of malaria. Lowered hemoglobin level would mean an increased oxidant stress, characterized by increased level of MDA but lowered ascorbate level. The reduced ascorbate is a measure of the deficit in the antioxidant generating system.

## 5. Conclusion

In conclusion, malaria disease progression increases MDA levels and decreases the ascorbate (vitamin C) and hemoglobin concentrations in children. It is recommended that future studies should investigate changes in other antioxidant vitamins like vitamins A and E and also higher number of subjects should be recruited into the study to validate some of the findings in this study. It is advisable to provide children with complicated malaria with vitamin C supplements, since they are susceptible to oxidative stress.

## Figures and Tables

**Table 1:** Ages and gender of study participants.

Variables	Control	Uncomplicated	Complicated	P value
	(n=15)	(n=51)	(n=17)	
*Age (years) (mean ± SD)*	3.80±1.66	4.32±2.81	4.27±2.96	0.801
*Age range of the wards (years)*				0.777
0-5	10 (66.7%)	35 (68.6%)	11 (64.7%)	
6-10	5 (33.3%)	14 (27.5%)	6 (35.3%)	
11- 12	-	2 (3.9%)	-	
*Gender*				0.676
Male	8 (53.3%)	24 (47.1%)	7 (41.2%)	
Female	7 (46.7%)	27 (52.9%)	10 (58.8%)	

SD = standard deviation, P value<0.05 = statistically significant.

**Table 2:** Comparison of hemoglobin, serum MDA, and vitamin C levels according to gender between uncomplicated and complicated malaria subjects.

Uncomplicated cases	Males (n=24)	Females (n=27)	p value
	mean±SD	mean±SD	
HB (g/dl)	10.3±1.7	10.7±1.6	0.391
MDA (nmol/ml)	4.8±1.4	5.1±1.9	0.458
Vitamin C (*μ*mol/L)	40.0±11.6	36.6±12.	0.310

Complicated malaria cases	Males (n=7)	Females (n=10)	p value
	mean±SD	mean±SD	

HB (g/dl)	7.6±1.5	7.0±2.5	0.311
MDA (nmol/ml)	5.6±1.3	5.0±2.1	0.233
Vitamin C (*μ*mol/L)	33.0±11.1	45.8±12.4	<0.001

P value<0.05 = statistically significant, HB = hemoglobin, MDA = malondialdehyde.

**Table 3:** Correlation analysis of MDA and vitamin C (ascorbic acid) against hemoglobin level among participants.

MDA		Vitamin C	
Pearson coefficient (r)	p value	Pearson coefficient (r)	p value
-0.119	0.650	0.201	0.433

P value<0.05 = statistically significant, MDA = malondialdehyde.

**Table 4:** Comparison ofhemoglobin, serum MDA, and vitamin C levels of control subjects and uncomplicated and complicated malaria.

Variables	Control (a)	Uncomplicated (b)	Complicated (c)	p value	Significant pairs
	(n=15)	Malaria (n=51)	Malaria (n=17)		
HB (g/dl)	10.70±1.65	10.50±1.64	7.25±1.91	**<0.0001**	a versus c (**<0.0001**)
MDA (nmol/ml)	4.62±1.85	4.50±1.58	6.68±0.70	**<0.0001**	b versus c (**<0.0001**), a versus c (**0.008**)
Vitamin C (*μ*mol/L)	49.57±21.80	40.53±13.22	38.17±12.33	**0.036**	a versus c (**0.019**)

P value<0.05 = statistically significant, HB = hemoglobin, MDA = malondialdehyde.

## Data Availability

The data used to support the findings of this study are included within the article.
